# Comparison of bacterial communities of conventional and A-stage activated sludge systems

**DOI:** 10.1038/srep18786

**Published:** 2016-01-05

**Authors:** Alejandro Gonzalez-Martinez, Alejandro Rodriguez-Sanchez, Tommaso Lotti, Maria-Jesus Garcia-Ruiz, Francisco Osorio, Jesus Gonzalez-Lopez, Mark C. M. van Loosdrecht

**Affiliations:** 1 Department of Civil Engineering, University of Granada, Campus de Fuentenueva, s/n, 18071, Granada, Spain; 2 Institute of Water Research, University of Granada, C/Ramón y Cajal, 4, 18071, Granada, Spain; 3 Department of Biotechnology, Technical University of Delft, Julianalaan 67,2628 BC, Delft, The Netherlands

## Abstract

The bacterial community structure of 10 different wastewater treatment systems and their influents has been investigated through pyrosequencing, yielding a total of 283486 reads. These bioreactors had different technological configurations: conventional activated sludge (CAS) systems and very highly loaded A-stage systems. A-stage processes are proposed as the first step in an energy producing municipal wastewater treatment process. Pyrosequencing analysis indicated that bacterial community structure of all influents was similar. Also the bacterial community of all CAS bioreactors was similar. Bacterial community structure of A-stage bioreactors showed a more case-specific pattern. A core of genera was consistently found for all influents, all CAS bioreactors and all A-stage bioreactors, respectively, showing that different geographical locations in The Netherlands and Spain did not affect the functional bacterial communities in these technologies. The ecological roles of these bacteria were discussed. Influents and A-stage bioreactors shared several core genera, while none of these were shared with CAS bioreactors communities. This difference is thought to reside in the different operational conditions of the two technologies. This study shows that bacterial community structure of CAS and A-stage bioreactors are mostly driven by solids retention time (SRT) and hydraulic retention time (HRT), as suggested by multivariate redundancy analysis.

The activated sludge process is the most common treatment of wastewater at municipal and industrial wastewater treatment facilities^1^. This technology can effectively eliminate many pollutants from wastewater with high efficiency and at reasonable costs. The so-called Adsorption-Belebungsverfahren (AB) process was developed in the 70’s as an energy efficient wastewater treatment process^2^. It is currently proposed as an essential element in an energy producing and efficient wastewater treatment process^3^,^4^,^5^.

The AB technology consists of a system of two bioreactors with an intermediate and a final clarifier after them, respectively. The first bioreactor, named A-stage, is highly loaded and mainly intended for organic matter removal; the second, named B-stage, is lowly loaded and mainly intended for nutrients removal. This technology does not need a primary sedimentation tank^6^. On the other hand, CAS consists of a one-bioreactor system, followed by a final clarifier. In this sense, the same processes of biological organic matter and nutrients removal are achieved in one bioreactor for CAS and in two bioreactors for the AB technology. AB systems have proved several advantages over conventional activated sludge (CAS) systems, such as lower energy consumption, lower reactor volume, and the capability to absorb better high variations in the influent characteristics^6^.

The hydraulic retention time (HRT) and sludge retention time (SRT) are two of the most important parameters in operation of bioreactors. They express the mean time that the fluid spends inside the bioreactor before becoming treated and the mean time that biomass spends inside the bioreactor after it is washed away with the effluent, respectively. In practical operation of bioreactors, variations of HRT and SRT are done in order to control the performance of bioprocesses.

The purpose of this work was to study the differences in bacterial community in CAS systems and AB systems. Influents and activated sludge of 10 full-scale plants - 7 with CAS technology and 3 with AB technology - were analyzed. This allowed to study the impact of the influent microbiome on the two processes. Given that bacterial community composition and diversity is thought to drive the performance of activated sludge systems^7^, differences between the CAS and AB systems are explained through bacterial community structure differences. Core genera for influent, CAS and A-stage samples were defined as follows: for the influent samples, genera with at least 1% relative abundance in at least 8/10 samples; for the CAS samples, genera with at least 1% relative abundance in at least 5/7 samples; for A-stage bioreactors, genera with at least 1% relative abundance in at least 2/3 samples.

## Results and Discussion

### Different configuration and operational conditions among systems studied: the CAS and the A-stage bioreactors

The different characteristics and operational conditions of the two technologies covered in this study (CAS and A-stage bioreactors) can be seen in Table 1. The most defining difference between these two technologies is the shorter HRT of A-stage bioreactors with respect to CAS. It is expected that microbial community structures of these bioprocesses respond to these different characteristics.

### Species richness, rarefaction and Hill diversity indices analysis

**Figure 1:**
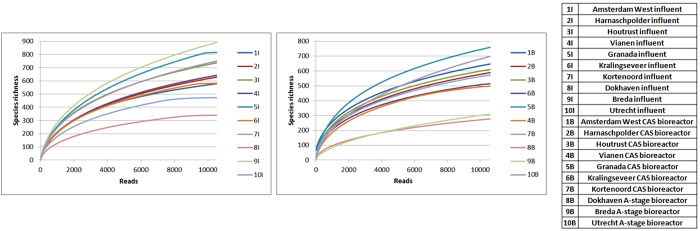
Rarefaction curves of influent samples (left) and bioreactor samples (right).

The rarefaction curves of influent and bioreactor samples are shown in Fig. 1. The calculated species richness, ACE, Chao1, Chao standard deviation, Shannon and Simpson indices of all samples are shown in Table 2. Overall, diversity and richness indices showed similar values among influent and A-stage bioreactor samples, while these values were higher for the CAS bioreactor samples. This suggests that diversity of bacterial communities is higher in CAS bioreactors than in influent samples or A-stage bioreactor samples.

### Bacterial community structure in influent and bioreactor samples: statistical analysis

**Figure 2:**
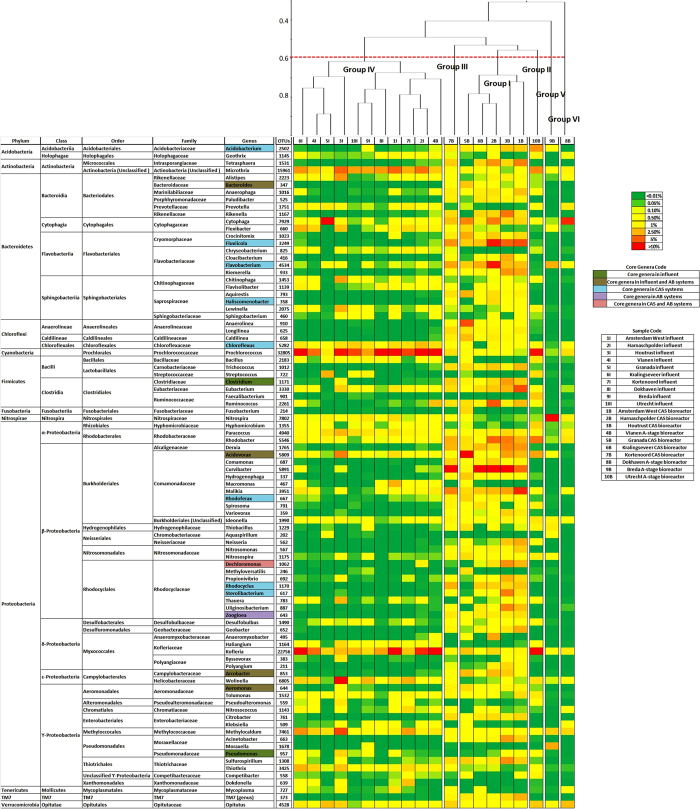
Phylogeny-dependent cluster analysis of all samples taking into account OTUs belonging to genera with >1% relative abundance in at least one of the samples (above) and heat map of all samples at genus level (below). The benchmark used for clustering groups definition is marked with a dashed line in the clustering tree.

Phylogeny-based cluster analysis of OTUs with >1% relative abundance in influent or bioreactor samples is shown in Fig. 2. Taking 0.6 as benchmark for differentiation^8^ we can cluster the samples in 6 groups. Group I comprises 5 CAS bioreactor samples, while Group III contains a single CAS bioreactor. Groups II, V and VI stand for single samples of an A-stage bioreactor. Group IV clusters all influent samples of CAS and AB systems, and a single CAS bioreactor sample. Non-phylogeny-based cluster analysis at class and genus level showed identical patterns than phylogeny-based cluster analysis (Fig. S1 and Fig. 2).

Phylogeny-based principal coordinates analysis of OTUs with >1% relative importance (Fig. S2) showed the same trend observed in phylogeny-based cluster analysis. There is a remarkable similarity among all CAS bioreactor samples, and also among all influents. A-stage bioreactor samples appear scattered and show uniqueness in similarity with all other samples. Samples 5I and 5B collected in Spain does not appear isolated and show similarity with samples collected in The Netherlands. Non-phylogeny-dependent principal coordinates analysis at class and genus level showed close similarity with the phylogeny-based principal coordinates analysis (Fig. S1 and S2).

It is shown that similarity exists among all influent samples regardless of the location they were collected. Bacterial composition of the feed has been suggested as a major factor in bioreactor bacterial community structure^9^, but given the similarity shown by all influent samples its impact on the variation between the different activated sludge processes studied can be considered negligible in this study. All CAS bioreactor samples are similar but scatter of A-stage bioreactor samples show that microbial diversity of these systems is much more unique than that of CAS bioreactors. Samples collected in Spain 5I and 5B showed a high similarity with samples collected in The Netherlands. Even though geographic location has been suggested as a major factor controlling microbial community structure^8^, it can be assumed that its impact in this study is negligible. We can say that, as a major hypothesis, the responsible for bacterial community structure in CAS and AB systems analyzed are the different operational parameters and wastewater treatment technology rather than the influent microbial community.

### Bacterial community structure of influent samples

In the phylogeny-based cluster analysis and principal coordinates analysis, all 10 influent samples were clustered in Group IV using the 0.6 benchmark. Regardless of the WWTP they feed, all influent samples have a similar bacterial community structure (Fig. 2). Similarities among bacterial community structure of all influents might be defined by wastewater characteristics and geographic proximity between all WWTPs sampled. All influents were composed by urban sewage coming from human use of wastewater and it is expected that all influents harbor similar communities, mostly coming from human gut. As well, it has been reported that bacterial communities in WWTPs tend to be similar for close geographical areas^8^.

At class level the dominance in all influents belong to β-Proteobacteria, ϒ-Proteobacteria, ε-Proteobacteria and Bacteroidia, although Clostridia was found to have a relatively high importance in a couple of the influent samples. Of 13 different major classes (>1% total abundance) appearing among all samples, 6 were found in all of them and 3 were represented in at least 7/10 of the samples. Only 4 classes - Actinobacteria, α-Proteobacteria, Chloroflexi and δ-Proteobacteria - were covered in 3/10 samples or less. These results are in accordance with previous studies^10^.

At family level, the most common taxonomic representation was that of Campylobacteraceae, Aeromonadaceae, Bacteroidaceae and Comamonadaceae, found at high relative abundance in all samples. 31 different families were identified among all influent samples. 6 families were found in all of them, and other 10 were represented in 7/10 samples and higher. 15 families were found in 3/10 of samples or lower, and all of these 15 accounted for relatively minor importance (<5% total abundance).

Bacterial community composition of all influent samples at genus level (>1% relative abundance) is represented in Fig. 2. A core of genera can be observed among the samples. *Aeromonas* (2.5–13%), *Arcobacter* (3–42%) and *Bacteroides* (5.5–19.5%) are present in all samples, *Acidovorax* (1–8%) and *Pseudomonas* (1–5%) are present in 9/10, and *Clostridium* (1–2.5%) in 8/10. These species comprehend both aerobic heterotrophs and fermenters. Among the core genera described, the genus *Clostridium* and *Bacteroides* have been reported to be two of the main consistent human gut bacteria^11^. In this sense, human gut bacteria are part of the core genera of influent wastewater in urban WWTPs.

### Bacterial community structure of CAS bioreactor samples

Cluster analysis and principal coordinates analysis showed the close similarity among all CAS bioreactor samples, clustered in Group III, Group IV and Group I (Fig. 2).

Group III is formed by a sample from Kortenoord CAS bioreactor without presettling. At genus level the heterotrophic, floc-forming *Haliscomenobacter* is the most represented (>11%). Other genera such as *Rhodocyclus* (>4.4%), *Rhodoferax* (>3.1%) and *Chloroflexus* (>3.9%) might play an important role for the system such as phosphorous removal, nitrogen removal and floc-backbone, respectively.

Sample 4B from Vianen WWTP is clustered within the Group IV, which also contains all influent samples. The most abundant genus in this bioreactor are *Arcobacter* (28%) and *Bacteroides* (25%), which together express more than 50% of total bacterial population inside the system. Other genera such as *Fluviicola* and *Aeromonas*, are represented at relatively low (<5%) abundance.

CAS systems at Vianen and Koortenord are operated without presettling, while all others have a primary sedimentation process. In this sense, the statistical deviation in clustering of samples 4B and 7B with respect to Group I might be caused by this fact.

Group I is formed by all other five CAS bioreactors. At class level the dominant are clearly the β-Proteobacteria, accounting for 16–30% relative abundance in all samples. Analysis of activated sludge systems through pyrosequencing has shown domination of Proteobacteria in activated sludge, with β-Proteobacteria found to be the dominant Proteobacteria in activated sludge systems in some of these studies^7^,^9^,^12^. Bacteroidetes also have a high importance in all samples. At genus level, 64 different genera were identified among all CAS samples in Group I (Fig. 2). Among these, *Acidobacterium* (~1–4%), *Chloroflexus* (~1.5–6.5%), *Flavobacterium* (~1–3.5%), *Rhodocyclus* (~1–3%) and *Rhodoferax* (~1.5–6.5%) were found in all samples, while *Dechloromonas* (~1–4%), *Fluviicola* (~1–4.5%), *Haliscomenobacter* (~3–16%) and *Sterolibacterium* (~1–1.6%) were found in 4/5 samples.

There are several genera shared by all CAS bioreactors, which might develop important roles for the functioning of CAS systems, as stated by Wang *et al.*^9^. *Rhodocyclus* was the only genus found in all 7 CAS bioreactors. These microorganisms are responsible for biological phosphate removal and all WWTPs investigated were designed as EBPR for this purpose. *Acidobacterium, Chloroflexus, Dechloromonas, Fluviicola* and *Rhodoferax* genera were found in 6/7 CAS bioreactors. Also, *Flavobacterium, Haliscomenobacter* and *Sterolibacterium* genera were found in 5/7 CAS bioreactors. *Acidobacterium* is thought to be responsible for BOD degradation in activated sludge systems^13^. *Fluviicola* species are ubiquitous in freshwater systems, respire only oxygen and develop colonies that form long filaments in rare occasions. Furthermore, metabolic machinery for degradation of complex organic compounds has been identified from the complete genome of *Fluviicola taffensis*^14^. The first *Sterolibacterium* species isolated came from a UASB reactor treating landfill leachate, and developed degradation of organic matter with oxygen or nitrate as terminal electron acceptor, with reduction of nitrate to dinitrogen^15^. In this way, *Acidobacterium, Sterolibacterium* and *Fluviicola* are the core genera members that carry out organic matter biodegradation in CAS systems. *Rhodocyclus* genus has been previously associated with N and P removal in WWTPs^16^. Species of *Rhodoferax* are thought to utilize nitrate as both electron acceptor and nitrogen source^17^ while *Dechloromonas* species have been reported for phosphorus removal and denitrification in WWTPs^7^. In this way, *Rhodocyclus, Rhodoferax* and *Dechloromonas*-related species are responsible for denitrification and biological phosphorous removal in CAS bioreactors. Also, *Sterolibacterium* could play an important role in nitrogen removal. *Chloroflexus*-related microorganisms have been found as backbone of floccular biomass in nutrient removal bioreactors in WWTPs^18^,^19^, and their role in the hydrolysis of proteins has been suggested^18^. *Haliscomenobacter* species were also found as backbone of floccular biomass^18^ and have been reported for the breakdown of N-acetylglucosamine^20^, thus able of scavenging decaying cell biomass. *Flavobacterium* have been found in WWTPs and have been reported to produce extracellular polymers that bound cells together^21^, thus they might act as floc-forming microorganisms.

None of the core genera defined for the CAS bioreactors have a nitrification metabolism. On the other hand, studies in 25 full-scale enhanced biological phosphorous removal (EBPR) wastewater treatment plants suggested the ammonium oxidizers *Nitrosomonas* and *Nitrosospira* and the nitrite oxidizer *Nitrospira* as core nitrifying genera^18^. In this study, *Nitrosomonas* genus was found in 6/7 of the CAS analyzed (~0.44–1.23%) and the genus *Nitrosospira* was found in only 1/7 of these bioreactors at 1.31% relative abundance (WWTP of Granada, sample 5B). The ammonium oxidizing genus *Nitrosococcus* was found in 3/7 of the CAS (~0.20–1.93%). While these ammonium oxidizing genera were not considered as core genera in CAS systems, the presence of these phylotypes is consistent among all CAS analyzed. These genera are responsible for the ammonium oxidation in these bioreactors. Also, the nitrite oxidizing genus *Nitrospira* was found in 6/7 CAS studied (~0.21–1.83%). As suggested by Nielsen *et al.*^18^, this genus is the responsible for nitrite oxidation in these activated sludge systems. The low contribution of the organisms in the microbial community is in accordance with the low growth yields of these autotrophic bacteria.

The bacterial communities found in these 7 CAS bioreactors showed many similarities with those presented in other studies regarding EBPR bioreactors in Denmark^18^. Notably, the presence and ecological roles of *Chloroflexus, Haliscomenobacter* and *Dechloromonas* suggested in this work are in accordance with those proposed by these authors. Major deviations in both ecological analysis were found on the biological phosphorous removing bacteria. Our pyrosequencing analysis showed that *Rhodocyclus* was the main phosphorous-removing bacteria in the 7 CAS bioreactors analyzed, while FISH techniques used by other authors showed that *Accumulibacter* was the main phylotype developing this ecological role in EBPR bioreactors^18^. Differences might reside in the geographical location, as it has been reported to impact bacterial community structure of activated sludge systems^8^, as well as differences in bioreactor technology and operational conditions. In this way, potential ecological roles in CAS bioreactors are shown in Table 3.

### Bacterial community structure of A-stage bioreactor samples

The three samples coming from A-stage bioreactors are not similar between them, all cluster separately in Groups, II, V and VI (Fig. 2).

Group II relates to a sample from Utrecht A-stage bioreactor. At class level dominance belongs to Bacteroidia class, with β-Proteobacteria relegated to a second role. This is different from CAS investigated in this study, where β-Proteobacteria were dominant in all cases. At genus level (>1%), *Bacteroides* and *Arcobacter* species are the most represented (~15%), with *Dechloromonas, Aeromonas, Geobacter* and *Clostridium* having a high relative abundance (~5%). *Bacteroides* and *Arcobacter* genera are aerotolerant, heterotrophic bacteria consistently found in urban WWTPs.

Group V includes only a sample from Breda A-stage bioreactor. At class level the majority of bacterial community is formed by β-Proteobacteria with an unprecedented abundance (>70%). At genus level the domination of the system belongs to *Hydrogenophaga* (>45%), with other genera being of relatively much lower importance, such as *Pseudomonas* or *Rhodoferax* (~11%). *Hydrogenophaga* has been identified as a heterotrophic bacterium which can utilize carbon under aerobic conditions and under anaerobic conditions through a denitrification metabolism^22^.

Group VI contains only a sample from Dokhaven A-stage bioreactor. At class level Flavobacteriia (~38%) and β-Proteobacteria (~29%) are the most represented, with ϒ-Proteobacteria having lower importance (~14%). At genus level, floc-forming *Flavobacterium* is the most represented with a high relative abundance (~32%). Genera such as *Zoogloea* (~10%), *Acidovorax* (~7%) and *Arcobacter* (~5%) are also of importance within the system.

In general, pyrosequencing analysis showed that bacterial community structure of A-stage bioreactors was clearly dominated by a few genera, leading to a low bacterial diversity of the bioreactors. Even though statistically all A-stage bioreactor samples were different in bacterial community structure, there existed some genera that were shared by all samples (Fig. 2). Being encountered in 2/3 samples were *Acidovorax* (2.5–7.5%), *Aeromonas* (3.3–6.0%), *Arcobacter* (1–25%), *Bacteroides* (4–23%), *Dechloromonas* (1–7%), *Hydrogenophaga* (3.6–46.1%) and *Rhodoferax* (1.1–11.6%), among others. The only genus found in all A-stage bioreactors sampled was *Zoogloea* (2–10%). Filamentous bacteria, such as *Zoogloea*, are thought to be crucial in the formation of flocular biomass in activated sludge systems^23^. A-stage bioreactors have a very short SRT and could be expected to have a population that is close to the influent microbial population. However this wasn’t the case indicating that the bioconversion of the soluble COD in the A-stage bioreactor is different from the sewer microbiome and specialized to the local conditions.

Following Wang *et al.*^9^, shared genera are found able to develop different important features for the functioning of activated sludge systems. A species of *Arcobacter* has been isolated from sewage sludge showing growth under aerobic conditions and poor growth under anaerobic conditions, with utilization of organic carbon and capability of nitrate reduction^24^. Species of *Acidovorax* have been reported from WWT systems being capable of aerobic, heterotrophic growth and of anaerobic growth through denitrification^25^. Strains of *Hydrogenophaga* isolated from activated sludge have been reported as putative heterotrophs^26^. *Aeromonas* have been found to produce chitin-degrading enzymes^27^, thus being capable of predation on cell biomass. Thus, *Arcobacter, Acidovorax, Aeromonas* and *Hydrogenophaga* genera, among others, state as core BOD-removal microorganisms in A-stage bioreactors. *Zoogloea* are thought to be capable of floccular biomass formation^28^. Environmental strains of *Bacteroides spp.* isolated from anaerobic digesters have shown heterotrophic metabolism and the capacity of forming extremely long filaments^29^. Therefore, presence of *Zoogloea* and *Bacteroides* species trigger floccular biomass formation in A-stage bioreactors. None of the core genera found in the A-stage samples were able to develop ammonium or nitrite oxidation. Ammonium oxidizing consistently found in activated sludge systems, such as *Nitrosomonas* or *Nitrosospira*, accounted for low relative abundance (0.33% maximum), as well as nitrite oxidizing *Nitrobacter* (below 0.01% in all A-stage samples). This is in line with the absence of nitrification in the A-stage bioreactor. Accordingly, *Dechloromonas, Acidovorax* and *Arcobacter* species are able to drive the denitrification taking place in A-stage bioreactors. Nitrate is supplied to these systems by effluent recirculation from the B-stage nitrifying bioreactor. In this way, potential ecological roles in A-stage bioreactors are shown in Table 3.

### Differences in bacterial community structure among CAS and A-stage bioreactors

#### Species richness

After pyrosequencing post-run analysis influent samples and bioreactor samples were cut to 10535 reads to provide the same sequencing depth for each sample to conduct further ecological analysis.

Species richness of samples was estimated through number of OTUs, ACE and Chao 1 estimators. Mean number of OTUs is greater for CAS bioreactor samples than for A-stage bioreactor samples, and mean ACE and Chao 1 richness estimators for CAS bioreactor samples are as well greater than that of A-stage bioreactor samples (Table 2). This is also confirmed by the rarefaction curves (Fig. 1).

Higher species richness in CAS bioreactors can be explained by the longer SRT and the presence of aerobic/anaerobic zones in these systems. Longer SRT benefits the proliferation of slow-growth microorganisms and consumption of a wide range of substrates. The long SRT also makes a cryptic growth cycle being relevant in these systems. In the A-stage bioreactor only very fast growing bacteria can maintain themselves and only the readily degradable BOD is converted. This likely associates with the lower species diversity.

#### Differences in core genera of CAS and A-stage bioreactors

Selection of different genera that carry out similar functions in activated sludge systems between CAS and A-stage bioreactors should be explained by differences in WWT technology, given the statistical insignificance of influent WW characteristics and geographical location. In fact, WWT system configuration has been proposed as way of selection for microbial communities thriving in these systems. In this case the difference in SRT can explain changes in bacterial communities among different types of WWT systems^12^.

Core genera identified for influent, CAS bioreactors and A-stage bioreactors are shown in Table 4. As well, the phylogenetic trees of CAS and A-stage are shown in the supplementary material as Figure S3 and Figure S4, respectively. As can be seen, several core genera of influent samples were also core genera in A-stage bioreactors, while influent samples shared none with CAS bioreactors core genera. Sameness of influent samples and A-stage bioreactors core genera is caused by the short SRT of the A-stage bioreactors. Thus, influent microbial community reaching the A-stage bioreactors has a short time to shift and therefore it leaves the bioreactor with small changes. On the contrary, the longer SRT in the CAS bioreactors impacts microbial community structure coming in with the influent. With sufficient time in the bioreactor, microbial community of the influent will decay (e.g. by protozoa predation) and will therefore not accumulate in the sludge. Accordingly, bacterial species that thrive on bacterial biomass accounted for 3.4–16.2% relative abundance in CAS and 3.3–6% in A-stage, respectively. Difference in relative abundance of *N*-acetylglucosamine utilizers implies that cell decay in CAS bioreactors is greater than in A-stage bioreactors.

#### Redundancy analysis of environmental variables and bacterial community structure

**Figure 3:**
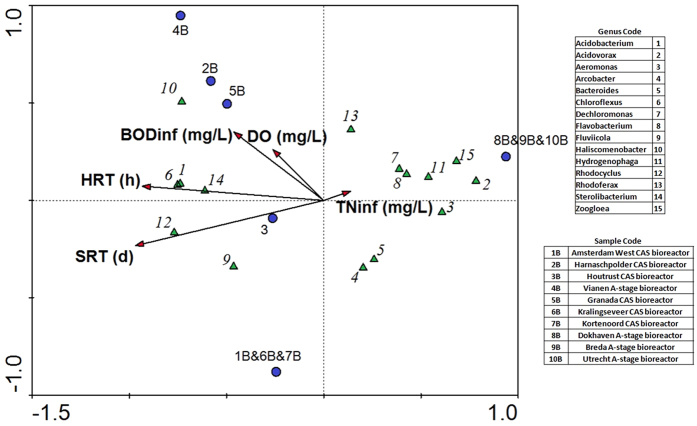
Multivariate redundancy analysis triplot of bioreactors pyrosequencing samples (1B–10B: circles), environmental parameters (dissolved oxygen concentration, BODinf, TNinf, HRT, SRT: arrows) and core genera of CAS and A-stage bioreactors analyzed (triangles).

RDA has been proven as a reliable method for the understanding of the relationship between microbial species and environmental parameters^30^. In this sense, an RDA expressing the relationship of bioreactor samples, their environmental parameters (influent BOD, influent nitrogen concentration, HRT, SRT, dissolved oxygen concentration and temperature) and relative abundance of CAS and A-stage core genera is shown in Fig. 3. The RDA showed that the most important variables explaining the ordination of the samples were the SRT and the HRT. As well, the RDA showed that the influence of temperature was negligible with respect to the composition of bacterial community structure. Also, the influence of dissolved oxygen concentration did not show a strong importance with the bacterial community composition. With the exception of *Arcobacter, Bacteroides* and *Haliscomenobacter*, all the 15 core genera were distributed in correlation with this variable. Interestingly, genera *Fluviicola, Rhodocyclus, Chloroflexus, Sterolibacterium* and *Acidobacterium* were correlated with positive HRT, while genera *Aeromonas, Acidovorax, Hydrogenophaga, Flavobacterium, Zoogloea* and *Dechloromonas* showed a clear relation with negative HRT. In this sense, the core genera of CAS bioreactors are correlated with positive HRT and SRT (with exception of *Flavobacterium*), and core genera of A-stage are correlated with negative values of HRT and SRT. RDA results suggested that CAS core genera increased their relative abundance as the HRT and SRT increases, while the contrary happens for the core genera of A-stage bioreactors’ core genera. In this way, statistical analysis supports the hypothesis that SRT and HRT are the factors that drive the different composition of bacterial core genera in the CAS and A-stage systems analyzed in this study. The negligible influence of temperature could be caused by the relatively slow contribution with respect to other operational parameters such as HRT or SRT.

Another RDA analysis for the differentiation of species within core genera was also constructed, and it is shown in Figure S5 in the supplementary material. Interestingly, some species within the same genus experienced differences in ordination with respect to the environmental variables temperature, dissolved oxygen, HRT, SRT, influent BOD and influent total nitrogen concentration. Remarkable differences existed among *Rhodoferax* and *Bacteroides* genera. In this sense, RDA showed that *Rhodoferax antarticus* is more favored than *Rhodoferax sp* at lower HRT, SRT and influent BOD. Also, *Bacteroides graminisolvens* dominated within its genus at higher HRT and SRT and lower influent BOD. All other genera showed that their belonging species were similarly affected by these environmental variables.

On the other hand, differences in bacterial community structure of CAS and A-stage bioreactors could also be driven by the influence of other operational variables. In this sense, temperature, conductivity and pH have been found to drive bacterial community structure of geographically distant WWTPs in China^9^, with especial relevance of temperature. In this study, the effects of temperature seemed to be hindered by the strong influence of other parameters such as HRT or SRT.

### Similarities in bacterial community structure of influent and A-stage bioreactor samples

Phylogeny-based cluster analysis and principal coordinates analysis of influent samples and A-stage bioreactor samples show that these two groups of samples are not similar in terms of >1% relative abundance OTUs assemblages (Fig. 2). Nevertheless, the bacterial core genera defined for influent samples and A-stage bioreactor samples shared significant similarities (Table 4). The four core genera *Acidovorax, Aeromonas, Arcobacter* and *Bacteriodes* were present in influent and A-stage bioreactor core genera in similar relative abundances. The similarity in core genera among these samples could be related to the SRT values in A-stage bioreactors. In general, the SRT in A-stage bioreactors is short. Moreover, conditions are more close to sewer conditions, the biodegradable COD in the reactor is still relatively high (i.e. no competition on substrate affinity, growth at maximal growth rate). In CAS, BOD is overall very low inside the reactor, i.e. competition on substrate affinity and not on growth rate. The A-stage resembles aerated sewer conditions more, so likely similar microbial genera will be active although the actual microbial species deviate due to the more aerated conditions

## Conclusions

The microbial community structure of ten different wastewater treatment systems and their influent were analyzed by high-throughput pyrosequencing. Seven of these were conventional activated sludge (CAS) systems, while the other three were A-stage stages of AB systems. Statistical phylogeny-based and non-phylogeny-based analyses showed that influents were similar in terms of microbial community structure, and the same holds for the different CAS systems analyzed. On the other hand, A-stage system samples showed statistical independence from themselves and other samples, showing that the bacterial communities of these bioreactors are very case specific. Several genera were found in all the samples for the influent, CAS or A-stage bioreactors. These genera were identified as core genera of these systems, and their ecological roles in urban wastewater treatment processes were hinted. The variability and uniqueness of the A-stage bioreactor microbiome likely result from the very high loading and growth rates applied in these systems, selecting for unique microbial communities as compared to the CAs and sewer systems. Multivariate analysis identified that HRT and SRT are the main operational parameters that drive the differences in bacterial core genera among the CAS and A-stage bioreactors analyzed.

## Materials and Methods

### Wastewater treatment plants characteristics

Ten activated sludge bioreactors were subjected to pyrosequencing analysis of their influent and bioreactor microbiota. Among these, seven were CAS and three were A-stage bioreactors. Nine of these bioreactors were located in The Netherlands, and one of them in Spain. The seven CAS bioreactors were configured as enhanced biological phosphorous (EBPR) bioreactors, with some of them presenting a presedimentation basin prior to activated sludge process. Characteristics and operational conditions of the bioreactors sampled in the study are shown in Table 1.

### Collection of biomass samples and DNA extraction

Sludge samples were collected by WWTP operators at the different plants, all of them following the same collection procedure. For each influent and each bioreactor, five points evenly distributed among its cross-sectional area and its volume, respectively, were chosen, and one sample of 200 mL was taken from each sample point. Sample harvesting and pretreatment for DNA extraction was done in accordance to Ni *et al.*^31^. For biomass collection, samples were centrifuged at 5000 rpm for 10 min at ambient temperature. Biomass was stored at −20 °C for future DNA extraction.Then five subsamples, one for each sampling point of each bioreactor, were treated as independent samples for DNA extraction purposes. 300 mg of pelleted biomass of each sample was collected for DNA extraction using the FastDNA SPIN Kit for Soil (MP Biomedicals, Solon, OH). The five DNA extracts of each bioreactor were then merged together for PCR tag-pyrosequencing^32^.

### PCR amplification and pyrosequencing

Forward primer 28F (5′-GAGTTTGATCNTGGCTCAG-3′) and reverse primer 519R (5′-GTNTTACNGCGGCKGCTG-3′)^33^ were used to amplify the 500 bp hypervariable regions V1–V3 of 16S rRNA gene of *Bacteria*^34^. Pyrosequencing was developed by Research & Testing Laboratory (Lubbock, Texas, USA) and followed the procedure described in Dowd *et al.*^35^. PCR amplification for pyrosequencing started with preheating at 94 °C for 3 minutes, then proceeded with 40 cycles of: 94 °C for 30 seconds; 60 °C for 40 seconds and 72 °C for 1 minute; amplification ended with an elongation step at 72 °C for 5 minutes.

### Pyrosequencing post-run analysis

Raw reads from pyrosequencing process were trimmed based on quality to eliminate poor-quality end reads. Quality trimming was done based on quality scores. Quality trimmed data was then clustered to clean particularly noisy reads. Using USEARCH^36^, seed sequences were provided and quality trimmed reads were clustered to them in a 4% divergence threshold, thus eliminating sequences that fail to encounter similar reads. Chimeric sequences were detected using *de novo* method implemented in UCHIIME^37^ over clustered data collected during the previous step. Denoising was then conducted to correct base pair errors and eliminate bad sequences. After denoising, a quality control screening was conducted in which sequences that did not meet quality criteria were eliminated. Quality criteria taken were 1) sequences with low quality tags (more than 1 error in barcode tag sequence) and 2) sequences shorter than 250 bp. Reads were then clustered into 0% divergence using USEARCH^37^ for taxonomy identification. A seed sequence for each cluster was then queried from a high-quality sequences database derived from NCBI using Kraken^38^ software that utilizes BLASTN+. Based upon the BLASTN+ identity, sequences were affiliated to different taxa levels as following: 1) at species level if divergence is less than 3%, 2) at genus level if divergence is 3–5%, 3) at family level if divergence is 5–10%, 4) at order level if divergence is 10–15%, 5) at class level if divergence is 15–20% and 6) phylum level if divergence is 20–23%. Sequences that fail to encounter queried sequences with less than 23% divergence were discarded. After this procedure the number of reads for influent wastewater samples ranged from 10535 to 22925, while for bioreactor samples ranged from 13227 to 28138, adding to a total of 283486 (140550 and 142936 for influent and bioreactor samples, respectively). For further analysis, influent wastewater samples and bioreactor samples were rarified and cut to 10535 for proper ecological comparison.

### Cluster analysis and principal coordinates analysis

Cluster analysis of amended samples was done for influent and bioreactor samples separately. Cluster analysis was developed a) at class level, b) at genus level and c) with phylogeny-dependent method. For the first two cases, sequences in each sample were merged to class and genus affiliation. Relative abundance of each class and genus, respectively, was used as weight for cluster analysis. Cluster analysis was based on Bray-Curtis dissimilarity and was conducted in R-Project. For phylogeny-dependent method the software Fast UniFrac^39^ was used. A reference tree that comprised all OTUs from genera with <1% total abundance level cutoff was generated utilizing pyrosequencing reads using MEGA 6.0 software^40^. A sample mapping file and a category mapping file were created following UniFrac tutorial (http://unifrac.colorado.edu/). Relative abundance was taken as weight for weighted phylogeny-dependent cluster analysis. Cluster analysis was conducted in UniFrac according to the instructions given in the software tutorial. Following the procedure developed by Zhang *et al.*^8^, the different samples were grouped after the 60% similarity in the cluster analysis, stating that samples belonged to the same group if they were clustered together past the 0.6 benchmark.

Principal coordinates analysis was developed for influent and bioreactor amended samples separately. Principal coordinates analysis was done a) at class level, b) at genus level and c) with phylogeny-dependent method. For the non-phylogeny-dependent method in a) and b), pyrosequencing reads were merged at class and genus levels, taking relative abundance as weight for principal coordinates analysis. Principal coordinates analysis was conducted using R-Project software. For phylogeny-dependent method Fast Unifrac^38^ was used. A reference tree that comprised all OTUs from genera with <1% total abundance level cutoff was generated utilizing pyrosequencing reads using MEGA 6.0 software^40^. Sample mapping file and category mapping file were developed as described above. Relative abundance of each OTU was also taken as weight for weighted principal coordinates analysis. Principal coordinates analysis was conducted in UniFrac according to the instructions given in the software tutorial.

### Heat maps

Heat maps of the microbial community at genus level of >1% for all samples were done for the characterization of the microbial community structure of all influents and bioreactors. Heat maps were developed using Microsoft Excel 2010.

### Species richness analysis and Hill diversity indices

ACE richness estimator and Chao1 richness estimator were calculated for all samples utilizing fossil package in R-Project software^41^. Rarefaction curves for all samples were calculated using the software aRarefactWin by S. Holland (University of Georgia, Athens; http://www.uga.edu/strata/AnRareReadme.html). Hill diversity indices of first order (Shannon-Wiener index) and of second order (Simpson index) were calculated for all samples using the package vegan implemented in R-Project.

### Phylogenetic analysis

A phylogenetic tree was made for the analysis of diversity of CAS bioreactor samples and A-stage bioreactor samples separately using MEGA 6.0 software^40^. Sequences obtained through pyrosequencing process were used for the study. These were phylogenetically related to close-similarity sequences in the GenBank database by BLAST searching. All sequences were then aligned using ClustalW alignment algorithm. The phylogenetic trees were calculated through the neighbor-joining statistical method, with test of phylogeny consisting on a bootstrap model of 1000 bootstrap replications and using the Jukes-Cantor substitution model, as has been previously done^42^.

### Redundancy analysis of environmental variables and bacterial community structure

Multivariate constrained redundancy analysis (RDA) was used to investigate the relationship between environmental parameters of the bioreactors analyzed in this study (influent BOD, influent nitrogen, HRT, SRT, dissolved oxygen and temperature) with the relative abundance of bacterial members at genus level in each of these bioreactors. Environmental variables were weighted by taking the decimal logarithm of their values plus 1. As well, the relative abundance of the core genera defined for both CAS and A-stage bioreactors was taken for this analysis. RDA was calculated through 499 unconstrained Monte Carlo simulations using the Canoco for Windows 4.5 software. Another RDA was developed to observe the organization of each of the species found for each of the core genera. This was done in the same way as previously described with the exception that the relative abundance of bacterial members at OTU level was taken for the analysis.

## Additional Information

**How to cite this article**: Gonzalez-Martinez, A. *et al.* Comparison of bacterial communities of conventional and A-stage activated sludge systems. *Sci. Rep.* **6**, 18786; doi: 10.1038/srep18786 (2016).

## Supplementary Material

Supplementary Information

## Figures and Tables

**Table 1:** Characteristics and operational conditions of bioreactors sampled in the study.

WWTP	Code	Country	Technology	HRT (h)	SRT (d)	BOD_inf_ (mg/L)	BOD_eff_ (mg/L)	TN_inf_ (mg-N/L)	TN_eff_ (mg-N/L)	MLSS (g/L)	TP_inf_ (mg-P/L)	TP_eff_ (mg-P/L)	Dissolved Oxygen (mg-O/L)	Temperature (°C)	pH
Amsterdam West	1	The Netherlands	CAS	17	-	262	< 5	53	10	5	9	1	1.5	20	7.2
Harnaschpolder	2	The Netherlands	CAS	27	25	343	< 5	33	1.5	4	6.7	<1	1.5	19	–
Houtrust	3	The Netherlands	CAS	15	14	350	< 5	46.8	8.4	4,2	8.1	1.1	1	16.2	–
Vianen	4	The Netherlands	CAS	35	27	212	2.2	47	3.4	4	6.5	1.3	1	15	7.0
Granada	5	Spain	CAS	23	–	449	20	75	9	3.7	16	1	2.5	22	7.5
Kralingseveer	6	The Netherlands	CAS	16	20	107	< 5	43	5.3	3.8	6	1.3	1.5	18.1	7.3
Kortenoord	7	The Netherlands	CAS	20	20	220	< 5	56	2.6	–	10	0.3	1.5	18.8	–
Dokhaven	8	The Netherlands	A-stage	0.7	0.27	170	< 5	44	12	–	6	1	1	18	7
Breda	9	The Netherlands	A-stage	0.41	0.60	207	< 5	47	24	2.5	6	3.4	0.2	18	7
Utrecht	10	The Netherlands	A-stage	0.89	0.42	157	< 5	45	10	–	7.6	-	0.2	18	7

All CAS plants have a presettling tank except Vianen and Kortenoord.

**Table 2:** Hill diversity indices of order 1 and order 2 (Shannon and Simpson index, respectively), ACE, Chao1 and Chao standard deviation (Chao SD) of all samples, letter “I” represent Influent samples, Letter “B” represents Bioreactor samples.

	1I	2I	3I	4I	5I	6I	7I	8I	9I	10I
**Shannon**	4.415	4.249	4.878	4.320	4.844	4.403	4.225	3.283	5.133	4.649
**Simpson**	0.961	0.9510	0.977	0.954	0.978	0.964	0.963	0.878	0.982	0.962
**ACE**	640.194	820.548	1038.800	903.701	980.148	664.993	533.928	390.813	1230.179	1079.984
**Chao1**	733.209	943.500	1160.961	1032.281	1254.806	788.079	670.750	493.782	1483.651	1247.112
**Chao SD**	16.011	19.260	18.295	19.117	31.488	19.336	21.639	19.145	28.746	23.033
	**1B**	**2B**	**3B**	**4B**	**5B**	**6B**	**7B**	**8B**	**9B**	**10B**
**Shannon**	5.061	4.772	4.915	4.621	5.137	3.964	4.783	3.663	2.831	4.488
**Simpson**	0.986	0.978	0.983	0.973	0.982	0.932	0.980	0.949	0.873	0.964
**ACE**	844.504	766.150	732.992	568.832	922.002	510.606	653.682	381.137	437.350	1188.606
**Chao1**	1000.869	885.045	841.079	638.847	1145.876	609.405	768.445	441.150	663.409	1395.003
**Chao SD**	22.004	19.110	18.272	14.177	27.075	17.848	19.125	14.362	33.242	25.268

Each number represent each wwtp following the code of the table 1.

**Table 3:** Potential ecological roles of core genera in CAS and A-stage bioreactors.

Genus	Bioreactor	BOD removal	Denitrification	P removal	Suspended biomass formation	Predation
Acidobacterium	CAS	Chen *et al.*^14^	–	–	–	–
Acidovorax	AB	Heylen *et al.*^26^	Heylen *et al.*^26^	–	–	–
Aeromonas	AB	Chong *et al.*^25^	–	–	–	Chong *et al.*^28^
Arcobacter	AB	Collado *et al.*^25^	Collado *et al.*^25^	–	–	–
Bacteroides	AB	Ueki *et al.*^30^	–	–	Ueki *et al.*^30^	–
Chloroflexus	CAS	-	-	–	Gonzalez-Gil & Holliger^20^	–
Dechloromonas	CAS & AB	Kim *et al.* 2013	Kim *et al.* 2013	–	–	–
Flavobacterium	CAS	-	–	–	Guo *et al.*^22^	–
Fluviicola	CAS	Woyke *et al.*^15^	–	–	–	–
Haliscomenobacter	CAS	Kragelund *et al.*^21^	–	–	Kragelund *et al.*^21^	Kragelund *et al.*^21^
Hydrogenophaga	AB	Kämpfer *et al.*^27^				
Rhodocyclus	CAS	-	Thomsen *et al.*^17^	Thomsen *et al.*^17^	–	–
Rhodoferax	CAS & AB	Yao *et al.*^19^	Yao *et al.*^19^	–	–	–
Sterolibacterium	CAS	Tarlera & Denner^16^	Tarlera & Denner^16^	–	–	–
Zoogloea	AB		–	–	Martins *et al.*^24^; Shao *et al.*^29^	–

**Table 4:**
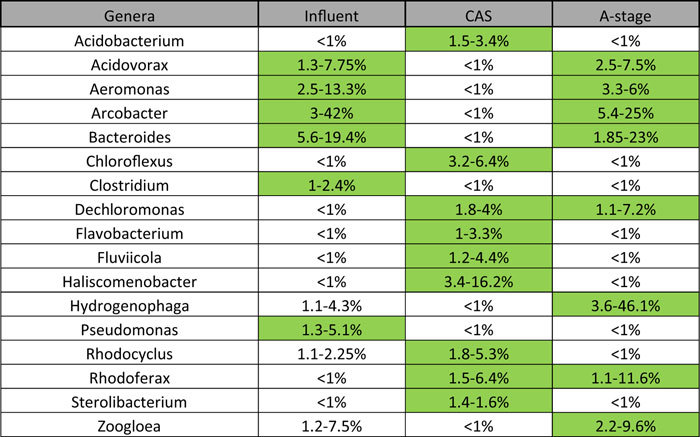
Shared genera in influent, CAS bioreactor and A-stage bioreactor samples (core genera are depicted in green)^41^.
