# Supplementary material for: Comparison of bacterial communities of conventional and A-stage activated sludge systems
Source: Sci Rep. 2016 Jan 5;6:18786. doi: 10.1038/srep18786 (PMC4700461; doi:10.1038/srep18786)

Comparison of bacterial communities of conventional and A-stage activated sludge systems

Alejandro Gonzalez-Martinez^1,*^, Alejandro Rodriguez-Sanchez^2^, Tommaso Lotti^3^, Maria-Jesus Garcia-Ruiz^1^, Francisco Osorio^1^, Jesus Gonzalez-Lopez^2^, Mark C. M. van Loosdrecht^3^

^1^ Department of Civil Engineering, University of Granada, Campus de Fuentenueva, s/n, 18071, Granada, Spain

^2^ Institute of Water Research, University of Granada, C/Ramón y Cajal, 4, 18071, Granada, Spain

^3^ Department of Biotechnology, Technical University of Delft, Julianalaan 67,2628 BC, Delft, The Netherlands

^*^ Corresponding author: A. Gonzalez-Martinez, Department of Civil Engineering, University of Granada, Campus de Fuentenueva, s/n, 18071 Granada, Spain; E-mail: [agon@ugr.es](mailto:agon@ugr.es); Phone: (+34) 958 244 170

**Figure S1 – Non-phylogeny dependent cluster analysis (top) and principal coordinates analysis (bottom) conducted at class level (a) and genus level (b).**


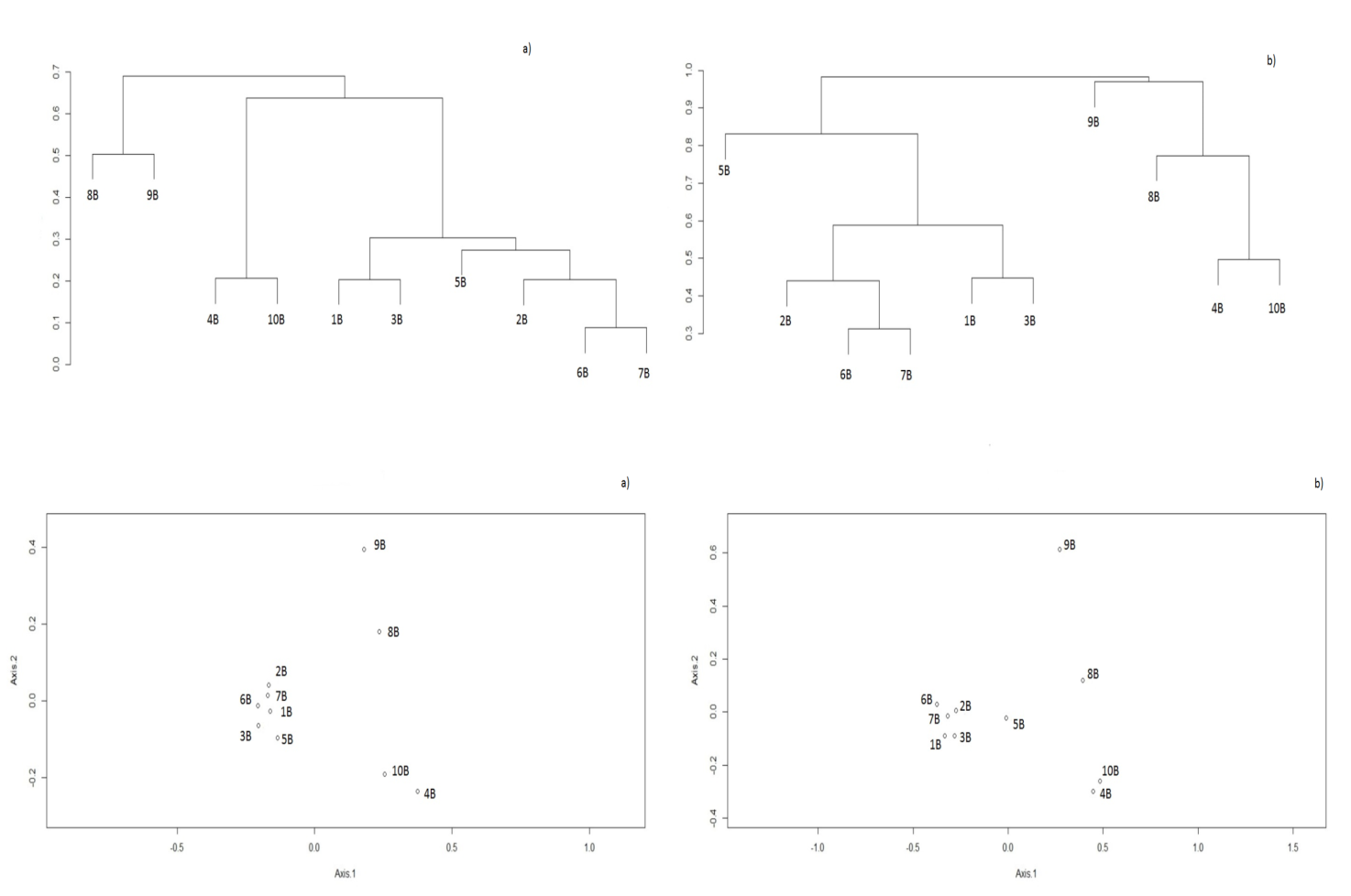


**Figure S2 – Phylogeny-based principal coordinates analysis of CAS influent and bioreactor samples (blue circles) and AB influent and bioreactor samples (red squares).**

**
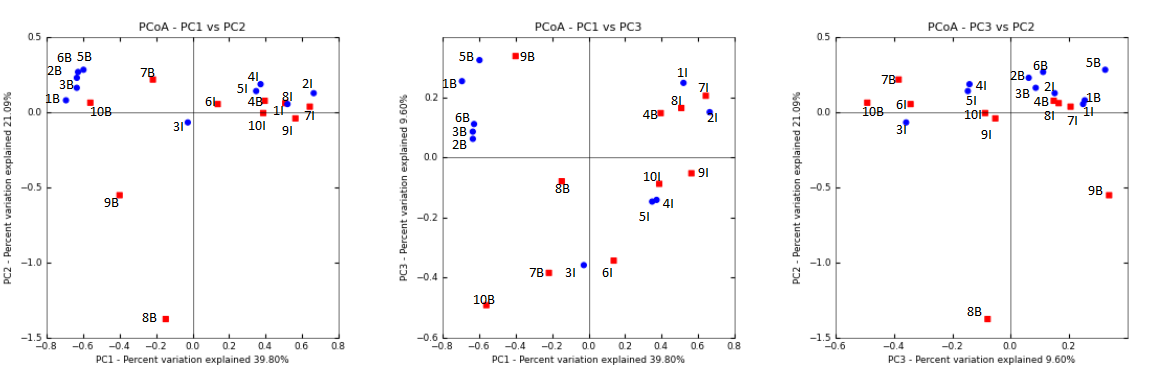
**


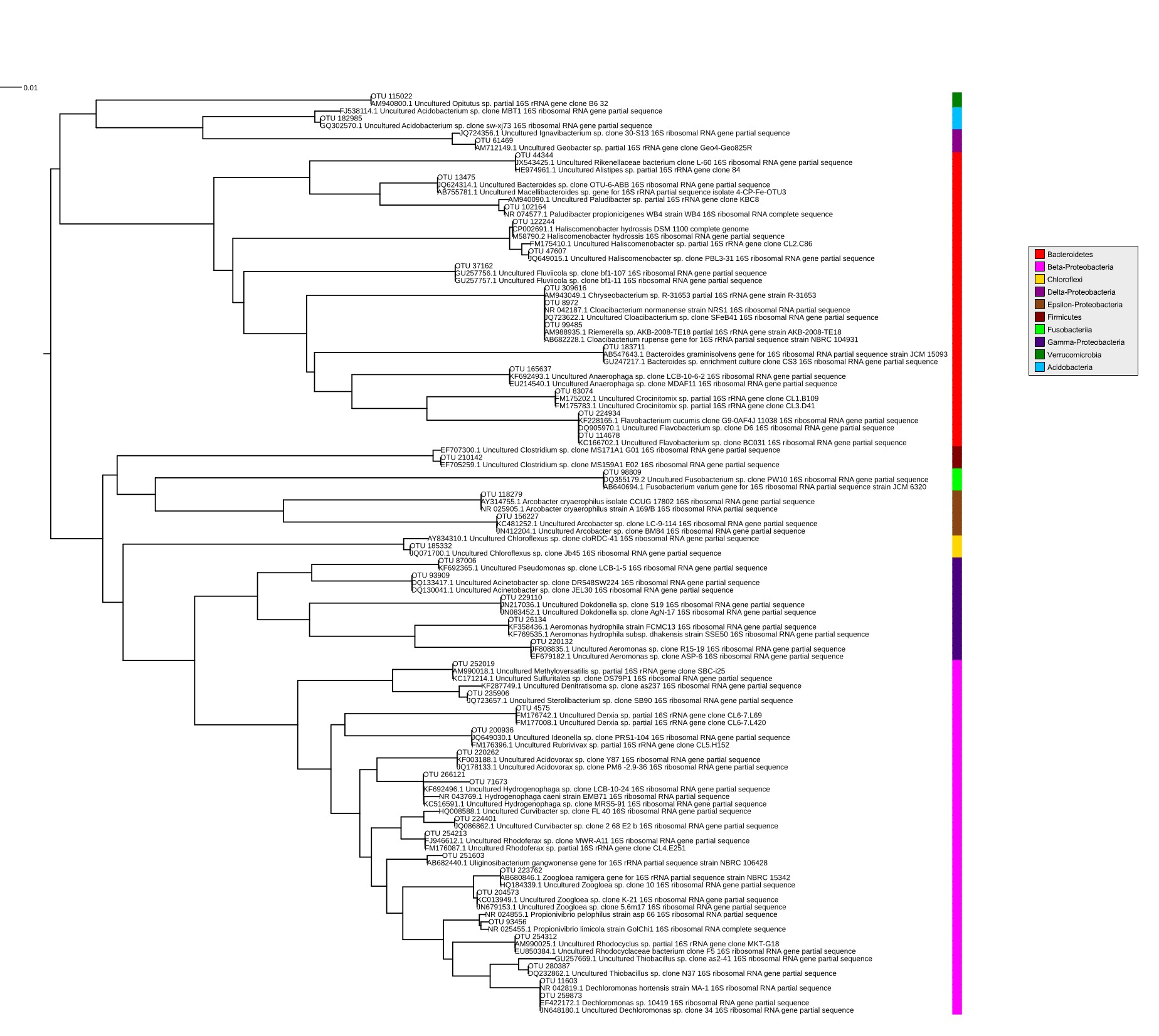
**Figure S3 – Phylogenetic tree of >1% relative abundance OTUs from all CAS bioreactors samples.**

**Figure S4 – Phylogenetic tree of >1% relative abundance of OTUs from all A bioreactors samples.**


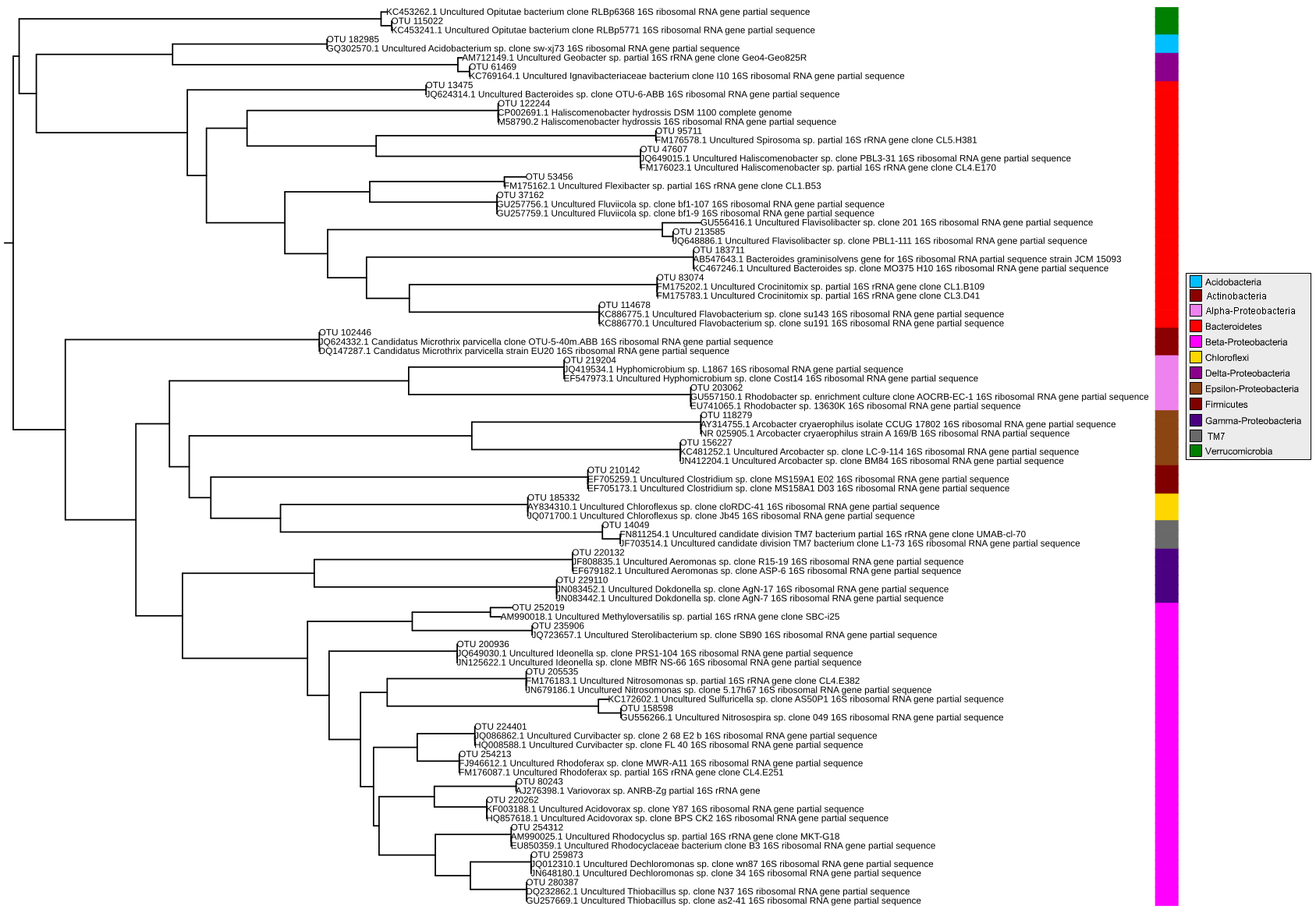


**Figure S5 - Multivariate redundancy analysis triplot of bioreactors pyrosequencing samples (1B-10B: circles), environmental parameters (dissolved oxygen concentration, BODinf, TNinf, HRT, SRT: arrows) and species among the core genera of CAS and A-stage bioreactors analyzed (triangles).**


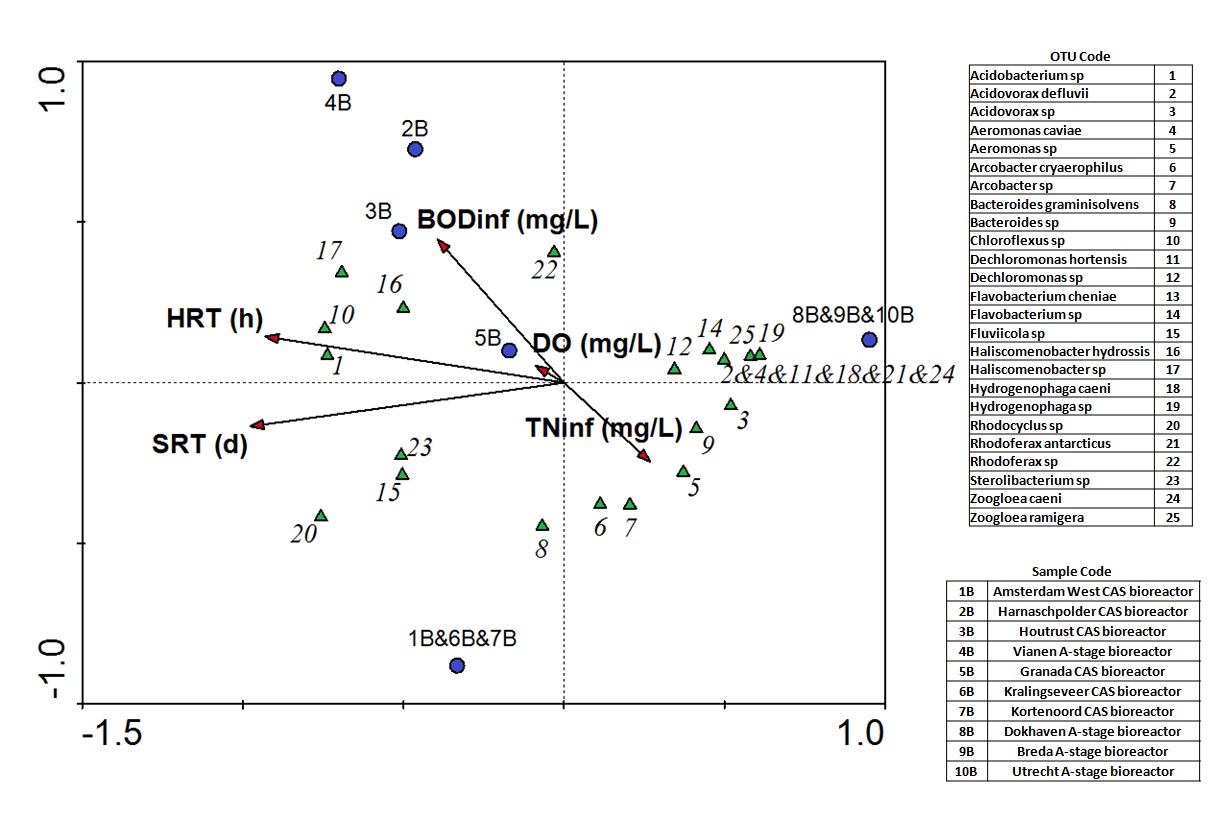

Supplement: Supplementary Information [file srep18786-s1.doc]
